# Sodium 2-mercaptoethanesulfonate monohydrate (coenzyme M sodium salt monohydrate)

**DOI:** 10.1107/S1600536808031814

**Published:** 2008-10-31

**Authors:** Stefan Mayr, Detlef Günther, Bernhard Jaun, W. Bernd Schweizer

**Affiliations:** a Laboratory of Organic Chemistry, ETH Zurich, Wolfgang-Pauli-Strasse 10, 8093 Zurich, Switzerland; b Laboratory of Inorganic Chemistry, ETH Zurich, Wolfgang-Pauli-Strasse 10, 8093 Zurich, Switzerland

## Abstract

The 2-thio­ethanesulfonate anion is the smallest known coenzyme in nature (HS–CoM) and plays a key role in methano­genesis by anaerobic archaea, as well as in the oxidation of alkenes by Gram-negative and Gram-positive eubacteria. The title compound, Na^+^·C_2_H_5_O_3_S_2_^−^·H_2_O, is the Na^+^ salt of HS–CoM crystallized as the monohydrate. Six O atoms form a distorted octa­hedral coordination geometry around the Na atom, at distances in the range 2.312 (4)–2.517 (3) Å. Two O atoms of the sulfonate group, one O atom of each of three other symmetry-related sulfonate groups plus the water O atom form the coordination environment of the Na^+^ ion. This arrangement forms Na–O–Na layers in the crystal structure, parallel to (100).

## Related literature

For related literature about HS–CoM, see: Allen *et al.* (1999 ▶); Bruchhausen *et al.* (1993 ▶); Günther & Hattendorf (2005 ▶); Graham *et al.* (2002 ▶); Latkoczy & Günther (2002 ▶); Mackay *et al.* (1999 ▶); Schramm *et al.* (1955 ▶); Thauer (1998 ▶). For the structure of the unhydrated Na HS–CoM salt, see: Bambagiotti-Alberti *et al.* (2007 ▶).
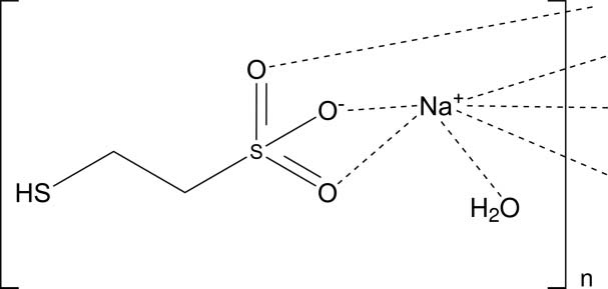


## Experimental

### 

#### Crystal data

Na^+^·C_2_H_5_O_3_S_2_^−^·H_2_O
*M* *_r_* = 182.19
Orthorhombic, 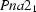

*a* = 23.4301 (8) Å
*b* = 5.0324 (2) Å
*c* = 6.1254 (2) Å
*V* = 722.24 (4) Å^3^
*Z* = 4
Mo *K*α radiation
μ = 0.74 mm^−1^
*T* = 223 K
0.26 × 0.20 × 0.01 mm

#### Data collection

Nonius KappaCCD diffractometer
Absorption correction: none
1647 measured reflections
1534 independent reflections
1263 reflections with *I* > 2σ(*I*)
*R*_int_ = 0.065

#### Refinement

*R*[*F* ^2^ > 2σ(*F* ^2^)] = 0.038
*wR*(*F* ^2^) = 0.131
*S* = 0.95
1534 reflections
88 parameters
1 restraint
H-atom parameters constrained
Δρ_max_ = 0.25 e Å^−3^
Δρ_min_ = −0.37 e Å^−3^
Absolute structure: Flack (1983 ▶), 627 Friedel pairs
Flack parameter: 0.13 (18)

### 

Data collection: *KappaCCD Server Software* (Nonius, 1997 ▶); cell refinement: *SCALEPACK* (Otwinowski & Minor, 1997 ▶); data reduction: *DENZO* (Otwinowski & Minor, 1997 ▶) and *SCALEPACK*; program(s) used to solve structure: *SIR97* (Altomare *et al.*, 1999 ▶); program(s) used to refine structure: *SHELXL97* (Sheldrick, 2008 ▶); molecular graphics: *PLATON* (Spek 2003 ▶); software used to prepare material for publication: *maXus* (Mackay *et al.*, 1999 ▶).

## Supplementary Material

Crystal structure: contains datablocks I, global. DOI: 10.1107/S1600536808031814/bh2190sup1.cif

Structure factors: contains datablocks I. DOI: 10.1107/S1600536808031814/bh2190Isup2.hkl

Additional supplementary materials:  crystallographic information; 3D view; checkCIF report

## Figures and Tables

**Table 1:** Selected bond lengths (Å)

Na9—O5^i^	2.312 (4)
Na9—O8^ii^	2.322 (4)
Na9—O7	2.404 (3)
Na9—O6^iii^	2.416 (2)
Na9—O5	2.456 (3)
Na9—O8	2.517 (3)

Symmetry codes: (i) 
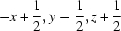
; (ii) 
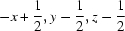
; (iii) 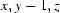.

**Fig. 1.**
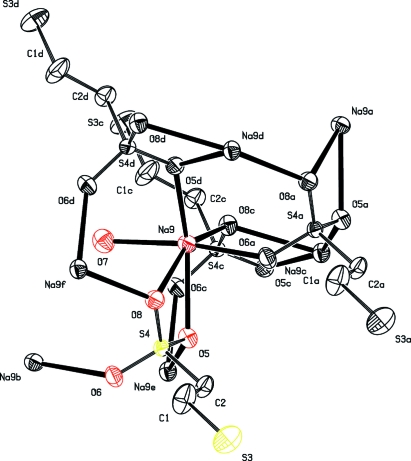
The molecular structure of sodium 2-thioethanesulfonate hydrate with 50% probability displacement ellipsoids. H atoms are omitted for clarity. The complete coordination of the Na atom is shown. Symmetry codes: (a) x, -1+y, z; (b) x, 1+y, z; (c) 1/2-x, -1/2+y, -1/2+z; (d) 1/2-x, -1/2+y, 1/2+z; (e) 1/2-x, 1/2+y, -1/2+z; (f) 1/2-x, 1/2+y, 1/2+z.

**Fig. 2.**
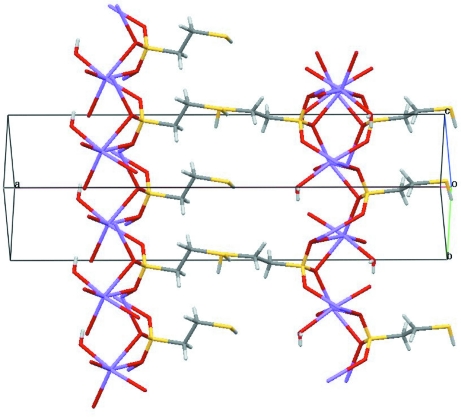
Crystal structure viewed down the diagonal of the a–b axis, showing the layer structure of Na—O clusters.

## supplementary crystallographic information

### Comment

The title compound includes 2-thioethanesulfonate ion, the smallest known coenzyme in nature, coenzyme M, which plays a key role in methanogenesis by anaerobic archaea (Thauer, 1998) and in the oxidation of alkenes by gram-negative and gram-positive eubacteria (Allen *et al.*, 1999). Furthermore its sodium salt (mesna) is medically used as mucolytics and to prevent urotoxic side effects of certain anticancer drugs (Bruchhausen *et al.*, 1993). Whereas the biosynthesis of coenzyme M starts by sulfitation of phosphoenolpyruvate (Graham *et al.*, 2002), the chemical synthesis begins from sodium 2-bromoethanesulfonate and thiourea in ammoniacal solution (Bruchhausen *et al.*, 1993). Since 2-thioethanesulfonic acid represents a highly viscous oil decomposing under release of hydrogen sulfide at room temperature, it is usually stored and sold as stable sodium or ammonium salt (Schramm *et al.*, 1955). The title compound is the monohydrate of the sodium salt.

Six O atoms show a distorted octahedral coordination geometry around the Na atom at distances in the range of 2.312 (4)–2.517 (3) Å (Fig. 1). Two O atoms of a SO_3_ group and one O of three other SO_3_ groups plus the water O atom form the coordination sphere of the Na^+^ ion. This forms Na–O–Na layers parallel to (100) in the crystal (Fig. 2).

The crystal structure of the unhydrated form (Bambagiotti-Alberti *et al.*, 2007; CSD refcode *UDUVUL*) shows a similar six-fold coordination of the Na atom where the water O atom is replaced by an O-atom of a SO_3_ group. The conformation of the S atoms is *antiperiplanar* in our compound and *gauche* in the unhydrated form.

### Experimental

When adding pure ethanol to a concentrated solution of 2-thioethanesulfonic acid in water, we noticed a precipitating white crystalline mass never described before in the literature. Micro elementary analysis based on the empirical formula (C_2_H_8_O_4_S_2_) of the hydrated acid (HS–CoM^-^H_3_O^+^) showed significantly low values for C and H. At the same time ^1^H and ^13^C NMR analysis of the precipitate in D_2_O ruled out any organic impurities. Investigations into the crystals by laser ablation inductively coupled plasma sector field mass spectrometry (LA-ICP-SF MS), however, clearly revealed the presence of sodium in hyperstoichiometric amounts: molar ratio (Na-23)/(S-32) = 1.34 (RSD: 8,5%, n = 9) (Günther & Hattendorf, 2005; Latkoczy & Günther, 2002). The white precipitate consisted of two different types of crystals, needles and thin plates. The needles were used for structure analysis by X-ray diffraction.

### Refinement

H-positions for the methylene CH_2_ groups have been calculated with fixed distance of 1.08 Å. H atoms for the water molecule and the thiol group have been taken from a difference map and were included in the refinement in their as-found positions.

### Crystal data

**Table:**

Na^+^·C_2_H_5_O_3_S_2_^−^·H_2_O	*D*_x_ = 1.676 Mg m^−^^3^
*M**_r_* = 182.19	Melting point: 473 K
Orthorhombic, *P**n**a*2_1_	Mo *K*α radiation, λ = 0.71073 Å
Hall symbol: P 2c -2n	Cell parameters from 5975 reflections
*a* = 23.4301 (8) Å	θ = 2.3–27.5°
*b* = 5.0324 (2) Å	µ = 0.74 mm^−^^1^
*c* = 6.1254 (2) Å	*T* = 223 K
*V* = 722.24 (4) Å^3^	Plate, colourless
*Z* = 4	0.26 × 0.20 × 0.01 mm
*F*(000) = 376.0	

### Data collection

**Table:**

Nonius KappaCCD diffractometer	*R*_int_ = 0.065
Radiation source: fine-focus sealed tube	θ_max_ = 27.5°, θ_min_ = 3.4°
CCD scans	*h* = −29→30
1647 measured reflections	*k* = −6→6
1534 independent reflections	*l* = −7→7
1263 reflections with *I* > 2σ(*I*)	

### Refinement

**Table:**

Refinement on *F*^2^	Primary atom site location: structure-invariant direct methods
Least-squares matrix: full	Secondary atom site location: difference Fourier map
*R*[*F*^2^ > 2σ(*F*^2^)] = 0.038	H-atom parameters constrained
*wR*(*F*^2^) = 0.131	*w* = 1/[σ^2^(*F*_o_^2^) + (0.1*P*)^2^] where *P* = (*F*_o_^2^ + 2*F*_c_^2^)/3
*S* = 0.95	(Δ/σ)_max_ < 0.001
1534 reflections	Δρ_max_ = 0.25 e Å^−^^3^
88 parameters	Δρ_min_ = −0.37 e Å^−^^3^
1 restraint	Absolute structure: Flack (1983), 627 Friedel pairs
0 constraints	Flack parameter: 0.13 (18)

### Fractional atomic coordinates and isotropic or equivalent isotropic displacement parameters (Å^2^)

**Table:**

	*x*	*y*	*z*	*U*_iso_*/*U*_eq_	
S3	−0.00830 (4)	0.7465 (2)	0.8298 (4)	0.0483 (4)	
H3	−0.0154	0.8542	0.6813	0.058*	
S4	0.17410 (3)	0.75424 (11)	0.66676 (19)	0.0185 (2)	
Na9	0.26442 (4)	0.3309 (2)	0.6658 (4)	0.0238 (3)	
O5	0.19813 (10)	0.6264 (6)	0.4720 (5)	0.0239 (7)	
O6	0.18238 (8)	1.0402 (4)	0.6710 (8)	0.0286 (5)	
O7	0.33586 (10)	0.6737 (5)	0.6717 (8)	0.0338 (5)	
H1	0.3259	0.8098	0.7595	0.023 (11)*	
H2	0.3374	0.7465	0.5332	0.047 (16)*	
O8	0.19475 (10)	0.6245 (6)	0.8649 (5)	0.0257 (7)	
C1	0.06798 (17)	0.8051 (9)	0.8424 (9)	0.0464 (11)	
H1A	0.0756	1.0166	0.8509	0.056*	
H1B	0.0846	0.7169	0.9900	0.056*	
C2	0.09943 (14)	0.6946 (6)	0.6517 (10)	0.0279 (8)	
H2A	0.0829	0.7827	0.5039	0.033*	
H2B	0.0920	0.4830	0.6431	0.033*	

### Atomic displacement parameters (Å^2^)

**Table:**

	*U*^11^	*U*^22^	*U*^33^	*U*^12^	*U*^13^	*U*^23^
S3	0.0235 (5)	0.0688 (8)	0.0526 (8)	−0.0031 (4)	0.0074 (6)	0.0024 (6)
S4	0.0182 (4)	0.0178 (4)	0.0194 (4)	−0.00146 (19)	0.0008 (4)	0.0002 (7)
Na9	0.0267 (6)	0.0228 (6)	0.0219 (6)	0.0034 (4)	−0.0009 (9)	−0.0004 (10)
O5	0.0302 (16)	0.0223 (15)	0.0191 (18)	−0.0013 (11)	0.0050 (14)	−0.0031 (12)
O6	0.0325 (10)	0.0188 (10)	0.0344 (12)	−0.0029 (8)	0.0011 (17)	−0.003 (2)
O7	0.0398 (13)	0.0345 (11)	0.0271 (13)	0.0012 (10)	0.004 (2)	−0.005 (2)
O8	0.0301 (16)	0.0271 (17)	0.0197 (18)	0.0020 (12)	−0.0011 (13)	−0.0027 (14)
C1	0.0228 (17)	0.063 (3)	0.053 (3)	−0.0050 (17)	0.010 (2)	−0.013 (3)
C2	0.0182 (14)	0.0364 (16)	0.029 (2)	−0.0033 (12)	−0.001 (2)	−0.008 (2)

### Geometric parameters (Å, °)

**Table:**

S3—C1	1.813 (4)	Na9—Na9^i^	4.0207 (5)
S3—H3	1.0714	Na9—Na9^ii^	4.0207 (5)
S4—O6	1.4522 (19)	Na9—Na9^v^	4.0207 (5)
S4—O8	1.460 (4)	O5—Na9^v^	2.312 (4)
S4—O5	1.468 (3)	O6—Na9^vi^	2.416 (2)
S4—C2	1.778 (3)	O7—H1	0.9012
S4—Na9	3.0028 (13)	O7—H2	0.9247
Na9—O5^i^	2.312 (4)	O8—Na9^iv^	2.322 (4)
Na9—O8^ii^	2.322 (4)	C1—C2	1.489 (6)
Na9—O7	2.404 (3)	C1—H1A	1.0800
Na9—O6^iii^	2.416 (2)	C1—H1B	1.0800
Na9—O5	2.456 (3)	C2—H2A	1.0800
Na9—O8	2.517 (3)	C2—H2B	1.0800
Na9—Na9^iv^	4.0207 (5)		
			
C1—S3—H3	96.1	O8—Na9—Na9^i^	83.64 (8)
O6—S4—O8	112.6 (2)	S4—Na9—Na9^i^	108.91 (6)
O6—S4—O5	113.4 (2)	Na9^iv^—Na9—Na9^i^	77.477 (11)
O8—S4—O5	110.64 (11)	O5^i^—Na9—Na9^ii^	113.78 (8)
O6—S4—C2	107.43 (13)	O8^ii^—Na9—Na9^ii^	35.38 (7)
O8—S4—C2	107.1 (2)	O7—Na9—Na9^ii^	125.28 (13)
O5—S4—C2	105.2 (2)	O6^iii^—Na9—Na9^ii^	59.83 (11)
O6—S4—Na9	127.52 (9)	O5—Na9—Na9^ii^	84.52 (8)
O8—S4—Na9	56.70 (12)	O8—Na9—Na9^ii^	128.86 (8)
O5—S4—Na9	54.35 (12)	S4—Na9—Na9^ii^	109.09 (5)
C2—S4—Na9	125.01 (11)	Na9^iv^—Na9—Na9^ii^	160.65 (6)
O5^i^—Na9—O8^ii^	106.77 (8)	Na9^i^—Na9—Na9^ii^	99.226 (14)
O5^i^—Na9—O7	92.45 (14)	O5^i^—Na9—Na9^v^	163.76 (8)
O8^ii^—Na9—O7	92.65 (14)	O8^ii^—Na9—Na9^v^	75.20 (9)
O5^i^—Na9—O6^iii^	91.25 (13)	O7—Na9—Na9^v^	71.31 (12)
O8^ii^—Na9—O6^iii^	93.87 (12)	O6^iii^—Na9—Na9^v^	104.78 (10)
O7—Na9—O6^iii^	171.25 (9)	O5—Na9—Na9^v^	31.43 (7)
O5^i^—Na9—O5	154.34 (14)	O8—Na9—Na9^v^	83.84 (8)
O8^ii^—Na9—O5	98.49 (14)	S4—Na9—Na9^v^	55.88 (5)
O7—Na9—O5	90.75 (11)	Na9^iv^—Na9—Na9^v^	99.226 (14)
O6^iii^—Na9—O5	82.52 (10)	Na9^i^—Na9—Na9^v^	160.65 (6)
O5^i^—Na9—O8	96.56 (14)	Na9^ii^—Na9—Na9^v^	77.476 (11)
O8^ii^—Na9—O8	156.12 (14)	S4—O5—Na9^v^	127.60 (18)
O7—Na9—O8	91.32 (12)	S4—O5—Na9	96.60 (16)
O6^iii^—Na9—O8	80.38 (10)	Na9^v^—O5—Na9	114.93 (10)
O5—Na9—O8	57.90 (7)	S4—O6—Na9^vi^	134.91 (12)
O5^i^—Na9—S4	125.55 (10)	Na9—O7—H1	112.0
O8^ii^—Na9—S4	127.54 (10)	Na9—O7—H2	107.3
O7—Na9—S4	88.94 (7)	H1—O7—H2	104.9
O6^iii^—Na9—S4	82.46 (6)	S4—O8—Na9^iv^	126.59 (19)
O5—Na9—S4	29.05 (8)	S4—O8—Na9	94.29 (16)
O8—Na9—S4	29.01 (8)	Na9^iv^—O8—Na9	112.33 (10)
O5^i^—Na9—Na9^iv^	74.00 (8)	C2—C1—S3	113.2 (3)
O8^ii^—Na9—Na9^iv^	162.52 (8)	C2—C1—H1A	108.9
O7—Na9—Na9^iv^	69.91 (11)	S3—C1—H1A	108.9
O6^iii^—Na9—Na9^iv^	103.59 (10)	C2—C1—H1B	108.9
O5—Na9—Na9^iv^	83.28 (8)	S3—C1—H1B	108.9
O8—Na9—Na9^iv^	32.29 (7)	H1A—C1—H1B	107.8
S4—Na9—Na9^iv^	55.67 (5)	C1—C2—S4	112.5 (3)
O5^i^—Na9—Na9^i^	33.64 (7)	C1—C2—H2A	109.1
O8^ii^—Na9—Na9^i^	113.20 (9)	S4—C2—H2A	109.1
O7—Na9—Na9^i^	123.68 (13)	C1—C2—H2B	109.1
O6^iii^—Na9—Na9^i^	58.49 (11)	S4—C2—H2B	109.1
O5—Na9—Na9^i^	129.85 (9)	H2A—C2—H2B	107.8
			
O6—S4—Na9—O5^i^	91.9 (3)	O8—Na9—O5—S4	4.61 (8)
O8—S4—Na9—O5^i^	−2.37 (19)	Na9^iv^—Na9—O5—S4	−16.81 (12)
O5—S4—Na9—O5^i^	−174.30 (19)	Na9^i^—Na9—O5—S4	50.69 (17)
C2—S4—Na9—O5^i^	−90.6 (3)	Na9^ii^—Na9—O5—S4	148.14 (13)
O6—S4—Na9—O8^ii^	−92.9 (3)	Na9^v^—Na9—O5—S4	−137.0 (2)
O8—S4—Na9—O8^ii^	172.8 (2)	O5^i^—Na9—O5—Na9^v^	147.7 (2)
O5—S4—Na9—O8^ii^	0.92 (18)	O8^ii^—Na9—O5—Na9^v^	−42.28 (14)
C2—S4—Na9—O8^ii^	84.6 (3)	O7—Na9—O5—Na9^v^	50.50 (18)
O6—S4—Na9—O7	−0.3 (3)	O6^iii^—Na9—O5—Na9^v^	−135.11 (15)
O8—S4—Na9—O7	−94.6 (2)	O8—Na9—O5—Na9^v^	141.6 (2)
O5—S4—Na9—O7	93.4 (2)	S4—Na9—O5—Na9^v^	137.0 (2)
C2—S4—Na9—O7	177.2 (2)	Na9^iv^—Na9—O5—Na9^v^	120.17 (13)
O6—S4—Na9—O6^iii^	178.0 (4)	Na9^i^—Na9—O5—Na9^v^	−172.34 (6)
O8—S4—Na9—O6^iii^	83.75 (18)	Na9^ii^—Na9—O5—Na9^v^	−74.88 (13)
O5—S4—Na9—O6^iii^	−88.17 (17)	O8—S4—O6—Na9^vi^	67.2 (4)
C2—S4—Na9—O6^iii^	−4.5 (3)	O5—S4—O6—Na9^vi^	−59.3 (4)
O6—S4—Na9—O5	−93.8 (3)	C2—S4—O6—Na9^vi^	−175.1 (4)
O8—S4—Na9—O5	171.92 (14)	Na9—S4—O6—Na9^vi^	2.7 (5)
C2—S4—Na9—O5	83.7 (3)	O6—S4—O8—Na9^iv^	1.0 (3)
O6—S4—Na9—O8	94.3 (3)	O5—S4—O8—Na9^iv^	129.03 (19)
O5—S4—Na9—O8	−171.92 (14)	C2—S4—O8—Na9^iv^	−116.9 (2)
C2—S4—Na9—O8	−88.2 (3)	Na9—S4—O8—Na9^iv^	122.0 (2)
O6—S4—Na9—Na9^iv^	65.9 (3)	O6—S4—O8—Na9	−121.06 (16)
O8—S4—Na9—Na9^iv^	−28.43 (14)	O5—S4—O8—Na9	7.01 (12)
O5—S4—Na9—Na9^iv^	159.65 (14)	C2—S4—O8—Na9	121.08 (15)
C2—S4—Na9—Na9^iv^	−116.6 (2)	O5^i^—Na9—O8—S4	178.06 (16)
O6—S4—Na9—Na9^i^	125.1 (3)	O8^ii^—Na9—O8—S4	−14.1 (4)
O8—S4—Na9—Na9^i^	30.82 (14)	O7—Na9—O8—S4	85.44 (17)
O5—S4—Na9—Na9^i^	−141.11 (14)	O6^iii^—Na9—O8—S4	−91.78 (16)
C2—S4—Na9—Na9^i^	−57.4 (2)	O5—Na9—O8—S4	−4.62 (8)
O6—S4—Na9—Na9^ii^	−127.6 (3)	Na9^iv^—Na9—O8—S4	132.6 (2)
O8—S4—Na9—Na9^ii^	138.14 (14)	Na9^i^—Na9—O8—S4	−150.81 (13)
O5—S4—Na9—Na9^ii^	−33.78 (14)	Na9^ii^—Na9—O8—S4	−54.07 (17)
C2—S4—Na9—Na9^ii^	49.9 (2)	Na9^v^—Na9—O8—S4	14.40 (12)
O6—S4—Na9—Na9^v^	−68.3 (3)	O5^i^—Na9—O8—Na9^iv^	45.45 (13)
O8—S4—Na9—Na9^v^	−162.63 (14)	O8^ii^—Na9—O8—Na9^iv^	−146.7 (2)
O5—S4—Na9—Na9^v^	25.45 (14)	O7—Na9—O8—Na9^iv^	−47.17 (17)
C2—S4—Na9—Na9^v^	109.2 (2)	O6^iii^—Na9—O8—Na9^iv^	135.61 (15)
O6—S4—O5—Na9^v^	−8.3 (3)	O5—Na9—O8—Na9^iv^	−137.2 (2)
O8—S4—O5—Na9^v^	−135.9 (2)	S4—Na9—O8—Na9^iv^	−132.6 (2)
C2—S4—O5—Na9^v^	108.8 (2)	Na9^i^—Na9—O8—Na9^iv^	76.58 (13)
Na9—S4—O5—Na9^v^	−128.7 (2)	Na9^ii^—Na9—O8—Na9^iv^	173.32 (6)
O6—S4—O5—Na9	120.40 (17)	Na9^v^—Na9—O8—Na9^iv^	−118.21 (13)
O8—S4—O5—Na9	−7.21 (12)	S3—C1—C2—S4	179.9 (2)
C2—S4—O5—Na9	−122.50 (16)	O6—S4—C2—C1	−60.6 (4)
O5^i^—Na9—O5—S4	10.8 (4)	O8—S4—C2—C1	60.6 (4)
O8^ii^—Na9—O5—S4	−179.26 (15)	O5—S4—C2—C1	178.3 (3)
O7—Na9—O5—S4	−86.47 (18)	Na9—S4—C2—C1	121.5 (3)
O6^iii^—Na9—O5—S4	87.92 (15)		

Symmetry codes: (i) −*x*+1/2, *y*−1/2, *z*+1/2; (ii) −*x*+1/2, *y*−1/2, *z*−1/2; (iii) *x*, *y*−1, *z*; (iv) −*x*+1/2, *y*+1/2, *z*+1/2; (v) −*x*+1/2, *y*+1/2, *z*−1/2; (vi) *x*, *y*+1, *z*.

## References

[bb1] Allen, J. R., Clark, D. D., Krum, J. G. & Ensign, S. A. (1999). *Proc. Natl Acad. Sci. USA*, **96**, 8432–8437. 10.1073/pnas.96.15.8432 PMC17533 10411892

[bb2] Altomare, A., Burla, M. C., Camalli, M., Cascarano, G. L., Giacovazzo, C., Guagliardi, A., Moliterni, A. G. G., Polidori, G. & Spagna, R. (1999). *J. Appl. Cryst.* **32**, 115–119.

[bb3] Bambagiotti-Alberti, M., Bruni, B., Di Vaira, M. & Giannellini, V. (2007). *Acta Cryst.* E**63**, o1796.

[bb4] Bruchhausen, F. V., Dannhardt, G. & Ebelet, S. (1993). Editors. *Hagers Handbuch der Pharmazeutischen Praxis*, Vol. 8, p. 890. Berlin, Heidelberg, New York, London, Paris, Tokyo, Hong Kong, Barcelona, Budapest: Springer.

[bb5] Flack, H. D. (1983). *Acta Cryst.* A**39**, 876–881.

[bb6] Graham, D. E., Xu, H. & White, R. H. (2002). *J. Biol. Chem.* **277**, 13421–13429. 10.1074/jbc.M201011200 11830598

[bb7] Günther, D. & Hattendorf, B. (2005). *TrAC Trends Anal. Chem.* **24**, 255–265.

[bb8] Latkoczy, C. & Günther, D. (2002). *J. Anal. At. Spectrom.* **17**, 1264–1270.

[bb9] Mackay, S., Gilmore, C. J., Edwards, C., Stewart, N. & Shankland, K. (1999). *maXus* Nonius BV, Delft, The Netherlands, MacScience Co. Ltd, Japan, and University of Glasgow, Scotland.

[bb10] Nonius (1997). *KappaCCD Server Software* Nonius BV, Delft, The Netherlands.

[bb11] Otwinowski, Z. & Minor, W. (1997). *Methods in Enzymology*, Vol. 276, *Macromolecular Crystallography*, Part A, edited by C. W. Carter Jr & R. M. Sweet, pp. 307–326. New York: Academic Press.

[bb12] Schramm, C. H., Lemaire, H. & Karlson, R. H. (1955). *J. Am. Chem. Soc.* **77**, 6231–6233.

[bb13] Sheldrick, G. M. (2008). *Acta Cryst.* A**64**, 112–122. 10.1107/S0108767307043930 18156677

[bb14] Spek, A. L. (2003). *J. Appl. Cryst.* **36**, 7–13.

[bb15] Thauer, R. K. (1998). *Microbiology*, **144**, 2377–2406. 10.1099/00221287-144-9-2377 9782487

